# The qualitative study of intentional self-harm in Thailand: Focusing on predisposing child - rearing environments and self-harm cessation

**DOI:** 10.1192/j.eurpsy.2023.2368

**Published:** 2023-07-19

**Authors:** N. Limsuwan

**Affiliations:** 1 Department of Psychiatry, Faculty of Medicine Ramathibodi Hospital, Mahidol University, Bangkok, Thailand

## Abstract

**Introduction:**

Intentional self-harm in adolescents and young people included both suicidal behaviors and nonsuicidal self-injury (NSSI) is a serious issue in mental health systems. However, the majority of studies on self-harm in adolescents and young people focused on quantitative methodology which might has limitations to explain this complex phenomenon of intentional self-harm.

**Objectives:**

This study aimed to describe the subjective experiences of adolescents and young people who presented with intentional self-harm in order to provide better understanding of this behavioral phenomenon.

**Methods:**

This is an exploratory qualitative study used phenomenological processes and thematic analysis.

**Results:**

Twenty subjects aged 13-29 years were included in this study. The results revealed 6 themes regarding predisposing child - rearing environments and 9 themes regarding factors related to cessation of intentional self-harm.

- **6 themes regarding predisposing child - rearing environments**

1. Lack of emotional responsiveness/emotional neglect
2. Negativity, criticism and harsh punishment
3. High academic expectations
4. Comparison with siblings
5. Superficial responsiveness
6. Enmeshment and over involvement

- **9 themes regarding factors related to cessation of intentional self-harm**

1. Negative perception to self-harm and desire to stop
2. Increase of adaptive coping
3. Finding life purposes
4. Improvement of psychiatric symptoms
5. Supportive relationships and verbalization
6. Treatments / interventions
7. Unwanted consequences of self–harm
8. Situations related to positive feelings
9. Behavioral control

Moreover, this study demonstrated the important functions of self-harm as an intrapersonal strategy for emotional regulation.

**Conclusions:**

This study underscored the importance to view self-harm as a complex phenomenon and it is essential to understand the developmental pathways as well as the pathways to cessation of these complex behaviors. Moreover, various internal and external factors related to cessation of intentional self-harm were demonstrated and verbalization in safe and supportive atmosphere tended to be an important process to promote the cessation or decrease of intentional self-harm.

**Disclosure of Interest:**

None Declared

